# Hypoxic Training for Judo: Practices, Perceptions and Education of Judo Athletes and Performance Staff

**DOI:** 10.3390/sports14070277

**Published:** 2026-07-03

**Authors:** Joshua Edward Till, Yoko Tanabe, Junsu Bae, Rafael Lima Kons, Ross Cloak, Andrew M. Lane

**Affiliations:** 1 Research Centre for Sport and Physical Activity, University of Wolverhampton, Walsall Campus, Walsall WS1 3BD, UK; r.cloak@wlv.ac.uk (R.C.); a.m.lane2@wlv.ac.uk (A.M.L.); 2 College of Law, Nihon University, Tokyo 101-8375, Japan; 3 Department of Physical Education, Yongin University, Yongin-si 17092, Republic of Korea; junsubae57@naver.com; 4 Department of Physical Education, Federal University of Bahia, Salvador 41170110, Brazil; rafakons0310@gmail.com

**Keywords:** judo, altitude training, hypoxic training, perceptions, strength and conditioning

## Abstract

Hypoxic training is widely used to enhance endurance performance, yet its application in combat sports such as judo is poorly understood. This study explored (1) hypoxic training practices, (2) perceptions, and (3) educational pathways among judo athletes and performance staff (coaches/practitioners). A total of 173 judo athletes and 39 performance staff completed an online questionnaire covering participant characteristics, hypoxic practices, education, and perceptions. Closed-ended responses were analysed using frequency statistics, and open-ended responses using thematic analysis. Hypoxic training was not widely used, with most respondents reporting no engagement. Among participants currently using hypoxic training, the primary aim was to enhance sea-level performance (13.7%), with limited use for altitude competition (8.5%). Natural altitude was the most common modality, with 7.1% respondents currently using it, typically at ≤1500 m. Only 20.8% of participants reported receiving or delivering education on hypoxic training. Perceptions were mixed, with 38.7% agreeing it benefits judo performance, although agreement was higher among performance staff than athletes. Thematic analysis identified perceived benefits (e.g., time-efficient fitness gains) and drawbacks (e.g., cost, access, and scheduling constraints). Hypoxic training is not a common practice in judo, but amongst some respondents it is perceived as potentially beneficial; these perceptions should not be interpreted as evidence of effectiveness. Its use is primarily oriented towards improving sea-level performance, and current knowledge appears largely informal. Greater sport-specific guidance and education may support more informed application in practice.

## 1. Introduction

Altitude training has been widely adopted since the success of high-altitude natives at the 1968 Mexico City Olympic Games (~2240 m) [1]. Traditionally, it was used to prepare athletes for competition at altitude through environmental acclimatisation [2,3,4]. More recently, research has shifted towards its use for enhancing sea-level performance [5,6,7], alongside emerging applications in clinical populations [8,9,10,11,12].

Altitude training (i.e., terrestrial/natural altitude) or simulated altitude training (i.e., chamber, tent, or mask-based systems) can be collectively referred to as hypoxic training. The use of mask-based systems, which involve connecting a mask and hose to a generator, should not be confused with the commercially available ‘altitude mask’, which is essentially a respiratory muscle trainer. Hypoxic training is proposed to induce both haematological (e.g., increased red blood cell volume and haemoglobin mass) [13,14,15] and non-haematological adaptations (e.g., increased oxidative enzyme activity, mitochondrial density, capillarisation, buffering capacity, and improved pH regulation) [16,17,18] which may enhance sea-level performance. Achieving these adaptations depends on the training model and hypoxic dose, which may be influenced by the mode of hypoxia (e.g., a greater severity of hypoxia required to elicit robust physiological responses when using simulated hypoxia compared with natural altitude). Traditional approaches such as live high–train high (LHTH) and live high–train low (LHTL) require prolonged exposure (>12 h·day^−1^) at moderate altitudes (~1800–2500 m) to stimulate erythropoiesis [18,19]. In contrast, intermittent hypoxic methods, including live low–train high (LLTH), involve shorter exposures (<3 h·day^−1^) and are less likely to elicit haematological adaptations, instead promoting peripheral muscular adaptations [19,20]. With the development in technology and growing accessibility in hypoxic training facilities, more innovative training solutions have become available for athletes. The rise in popularity of LLTH in intermittent sports, in particular, has witnessed the development of advanced training modalities such as high-intensity conditioning and resistance training performed in hypoxia [19,20]. In contrast to traditional means of training at altitude, notably endurance training, these modalities offer some unique benefits to athletes performing in intermittent sports (e.g., rugby), such as improved repeat-sprint ability [21]. Accordingly, practitioners should select training models based on the specific needs of the athlete, sport, and physiological adaptations targeted.

Hypoxic training has been widely studied in endurance [22,23] and, more recently, team sports [19,24], but remains under-researched in combat sports such as judo [25,26,27,28]. This gap may not reflect a lack of practitioner expertise, but rather the difficulty of translating principles developed in continuous, locomotor-based sports to the intermittent, high-intensity, weight-category demands of judo [29,30].

Judo is a grappling-based combat sport characterised by repeated high-intensity movements, including throws, submissions and hold-downs [31]. A standard match is set for 4 min; however, this could finish much sooner or can go on for several minutes into a period of sudden death, known as “golden score”. Therefore, high involvement of the oxidative energy system can be observed during a bout and is reflected by the high work-to-rest ratios (2:1–3:1) reported in the literature [29]. In support of this, Julio et al. (2017) [32] suggested that the oxidative energy system contribution (~70%) was predominant throughout a simulated judo match compared with the ATP-PCr (~21%) and glycolytic (~8%) energy systems, which demonstrated a greater contribution earlier on within the match. Despite this, high blood lactate values (10.4–25.1 mmol/L) have also been reported post-bout [33,34,35], suggesting substantial involvement of the glycolytic energy system. In addition to energy system development, judo athletes must also be able to produce high levels of strength and power. For example, performance in the countermovement jump demonstrated a strong positive association (r = 0.74; *p* < 0.01) with performance in the Special Judo Fitness Test (SJFT) [36]. Furthermore, grip strength-endurance has previously been suggested to discriminate between athlete levels, with elite athletes performing a greater number of repetitions (12 ± 5 vs. 9 ± 4 reps, respectively) in a dynamic judo-gi strength test compared with non-elite athletes [37]. Finally, in youth athletes, standing long jump performance and hand grip strength were able to predict close to 30% of the variance in performance in the SJFT [38]. Therefore, holistic development of all of the above fitness qualities should be a key focus for the competitive judo athlete.

The use of hypoxic training may be a valuable tool for improving the aforementioned fitness qualities in judo athletes. For instance, an innovative hypoxic training modality known as repeated-sprint training in hypoxia (RSH) (i.e., short all-out effort sprints with incomplete recovery) has demonstrated improved power output and fatigue resistance during a repeat-sprint ability (RSA) test compared with similar training completed at normoxia (i.e., sea-level) in athletes from various sports [39,40,41]. In support of this, Woorons et al. (2024) [42] investigated the effects of repeated-sprints completed under voluntary hypoventilation at low-lung volumes (VHL) (i.e., maximal end-expiratory breath holds) in elite judo athletes. The authors found that the VHL group improved their mean power output during the second half of the RSA test, along with a lower sprint decrement score, suggesting improved performance compared with the unrestricted breathing group, who did not observe these benefits. From a strength and power perspective, a few studies have explored resistance training in hypoxia (RTH) in judo athletes and have reported improved performance during a vertical jump task [25], with some research suggesting improvements in performance may be attained earlier than similar training conducted at sea level [27]. However, these findings should be interpreted with caution, as other research has found RTH to induce negative effects for peak knee extensor torque compared with resistance training in normoxia, which the authors suggested may be a product of greater peripheral fatigue from the addition of hypoxia [26]. Additionally, due to the limited number of studies exploring hypoxic training in judo, establishing whether hypoxic training is as effective as similar training at sea-level is yet to be determined. In addition to the training modality conducted in hypoxia, the mode of hypoxia and training model should also be considered for the judo athlete. As previously discussed, the traditional benefits associated with hypoxic training being primarily related to improved oxygen transport, may have some advantages for judo athletes given the bioenergetic demands of the sport. However, more experimental research is required to support this notion. Furthermore, these classical approaches to training often require a prolonged stay at terrestrial altitude, which is associated with some potentially adverse effects for athletes. For example, acute dehydration [43], increased risk of altitude-related illness [44] and a greater occurrence of sleep disturbance [45] are just a few risks associated with high-altitude exposure/training. Given the importance of recovery in judo, particularly when completing hard training sessions, making weight for competition and during high-volume training phases, some of these effects may be extremely detrimental to judo athletes. Therefore, coaches and practitioners should consider these factors when attempting to plan a hypoxic training phase for their athletes.

In the absence of sport-specific frameworks, coaches and practitioners are likely to rely on experiential knowledge, cross-sport analogies, and contextual constraints when implementing hypoxic training. However, these practices, underlying rationales, and associated perceptions have not yet been systematically documented.

Turner et al. (2019) [46] examined altitude training practices and perceptions among elite endurance athletes and support staff using an online questionnaire, providing insight into how altitude training is implemented and perceived in that context. Generally, positive responses were observed in the study by Turner et al. (2019) [46], which is in accordance with the breadth of literature supporting the use of hypoxic training for endurance athletes [22,23]. However, given the scarce number of studies exploring hypoxic training for the sport of judo [25,26,27,28] and the outlined differences in physical and physiological attributes, response rate and perceptions may vary from Turner’s (2019) [46] study. Despite these expected differences, adopting a similar approach in judo may help address the current gap in the literature by documenting whether hypoxic training is used in practice, how hypoxic training is used, understood, and taught within the sport.

Therefore, the aims of this study were threefold: (1) to examine hypoxic training practices among judo athletes and performance staff (coaches/practitioners); (2) to explore their perceptions of its use and (3) to investigate associated educational pathways. It is expected that hypoxic training practices applied by athletes and performance staff will be based on experiential knowledge from practitioners and coaches and may reflect traditional training recommendations adhered to by endurance and team-sport athletes, given the availability in the literature for those sports. It is also expected that athletes and performance staff will display positive perceptions towards hypoxic training as an effective training strategy. Finally, it is expected that education and understanding around the topic of hypoxic training will reflect experiential knowledge of athletes and performance staff. Given the absence of judo-specific frameworks, this study did not seek to identify optimal practice, but rather to document current practices, perceptions, and education in applied settings. Exploring the differences between judo athletes and performance staff responses will provide a valuable insight into the variability between key stakeholders responsible for implementing this type of training, compared with athletes who receive the training. Differences in practices, perceptions and education may highlight some key gaps in knowledge and understanding whereby educational intervention and evidence-based training recommendations can be developed through rigorous experimental research and applied to enhance training practices in the sport. It is worth noting that this study is strictly explorative and does not intend to evaluate the effectiveness of hypoxic training for judo athletes.

## 2. Methods

### 2.1. Study Design

This study used a cross-sectional research design in the form of a survey to examine hypoxic training practices, perceptions, and educational pathways among judo athletes and coaches/practitioners (hereafter referred to as performance staff). An anonymous online questionnaire was distributed via professional networks (e.g., British Judo, International Judo Federation), using the latest publicly available versions of social media in 2024/2025 (Instagram, LinkedIn, X), and in-person recruitment. Thus, to gain responses, this study utilised convenience sampling for participant recruitment. Data was collected from January 2024 to January 2025.

### 2.2. Participants

243 people responded to the questionnaire, 30 were excluded due to being under the age of 18 years old and an additional person was excluded as they admitted they had never performed judo. Therefore, 212 participants (Table 1) were included in the analysis. Due to the sampling strategy used (i.e., convenience sampling), this study did not use a priori sample size calculation. The inclusion criteria for this study were (a) a current or previous judo athlete or performance staff member and (b) 18 years old or over. This study was approved by the University of Wolverhampton ethics committee.

### 2.3. Procedure

The questionnaires used in this study were adapted from previous research on endurance athletes [46] and were administered via Google Forms (Google Forms, Google LLC, California, CA, USA). To account for differences in terminology, two separate questionnaires were developed, one for athletes and one for performance staff (Supplementary File S1). The questionnaires were translated into Portuguese and Korean, which were linguistically validated by two independent native-speaking researchers and distributed locally amongst these populations. To gain additional responses, the questionnaires were also translated into several other languages (Japanese, French, Italian, Spanish, German, Russian, Arabic, Chinese, Georgian and Hindi) to gain additional responses using the purchasable multilingual feature in Google Forms. After being reviewed by each member of the research team, the questionnaires were piloted on four performance staff members and four athletes from the British Judo National team to support face validity, readability, and contextual relevance. Participants involved in pilot testing were asked to provide feedback on question content, order, readability, visual appeal and time to completion. This resulted in minor adjustments to the wording and order of questions. No formal validation of the questionnaire was performed.

The final questionnaire comprised 28 questions for performance staff and 29 for judo athletes, as one question was irrelevant for staff to answer. Questions were divided into five sections: (1) informed consent; (2) participant information; (3) hypoxic training practices; (4) hypoxic training education and understanding and (5) hypoxic training perceptions. The questions were comprised of closed-ended and open-ended questions. A 4-point (i.e., “Never used”, “Previously used but not anymore”, “Currently used” and “Not used but would consider future use”) and 5-point (i.e., “Strongly disagree”, “Disagree”, “Neither agree nor disagree”, “Agree” and “Strongly agree”) Likert scale were used for some of the questions in the first and last section of the questionnaire as opposed to larger scales (e.g., 7-point) which are suggested to be harder to understand and are often misinterpreted [47]. Some responses were combined during the analysis, for example, “Agree” and “Strongly agree” to indicate overall agreement. Open-ended questions were answered by all participants regardless of whether they had experience using hypoxic training or not. Responses from participants who had not experienced hypoxic training represent their beliefs about hypoxic training rather than reflecting their practical experience. The questionnaires were accessed via a link on a cover letter (Supplementary File S2) that was emailed out to prospective participants through British Judo and the International Judo Federation (IJF). To gain additional respondents, the questionnaire was also sent out through International connections (i.e., Brazil, Japan, and Korea), advertised through social media platforms (e.g., Instagram, LinkedIn, and X) (Supplementary File S3) and distributed in person. Questionnaires that were completed in Portuguese, Korean and Japanese were back-translated into English upon receipt by three native-speaking researchers to ensure a consistent meaning of the content. The survey was assessed against the Checklist for Reporting Results of Internet E-Surveys (CHERRIES) [48] and Checklist for Reporting of Survey Studies (CROSS) [49] (Supplementary Files S4 and S5, respectively).

### 2.4. Statistical Analyses

Questionnaire responses were exported to Microsoft Excel (Microsoft Excel, Version 16.0, Microsoft, 2016, Redmond, WA, USA) for data cleaning (e.g., removing data input faults) and preparation. Closed-ended responses were analysed in SPSS (IBM SPSS Statistics, Version 25.0, IBM Corporation, Armonk, NY, USA) using descriptive statistics, with frequency distributions reported as percentages. Open-ended responses were analysed in NVivo (NVivo, Version 14.0, Lumivero, Denver, CO, USA) using reflexive thematic analysis following the six-phase approach outlined by Braun and Clarke (2006) [50]: data familiarisation, code generation, theme development, theme review, theme definition, and reporting.

## 3. Results

### 3.1. Participant Geolocation

Participants were recruited from multiple countries, predominantly from East Asia, South America and Europe (Table 2).

### 3.2. Hypoxic Training Practices in Judo

**Figure 1:**
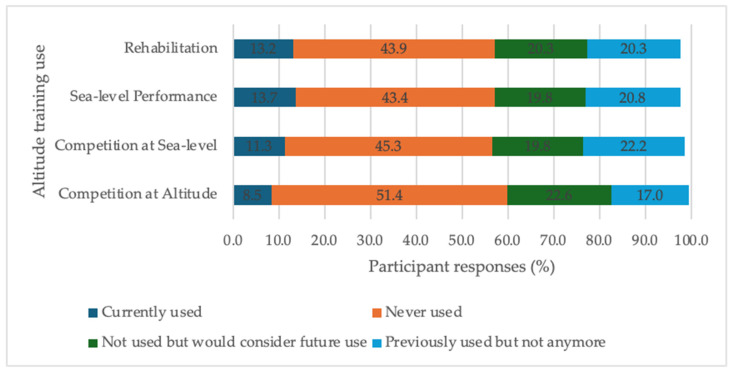
Participant response rate for different uses of hypoxic training.

Overall, the use of hypoxic training was low across the sample. As there was no overall item indicating whether participants had previously experienced hypoxic training or not, the following values are derived from specific items and do not represent a single overall percentage of users. Among those reporting use, the primary purposes were to enhance sea-level performance (13.7%) and support injury rehabilitation (13.2%), with less use for preparing for competition at sea level (11.3%) or at altitude (8.5%) (Figure 1).

**Figure 2:**
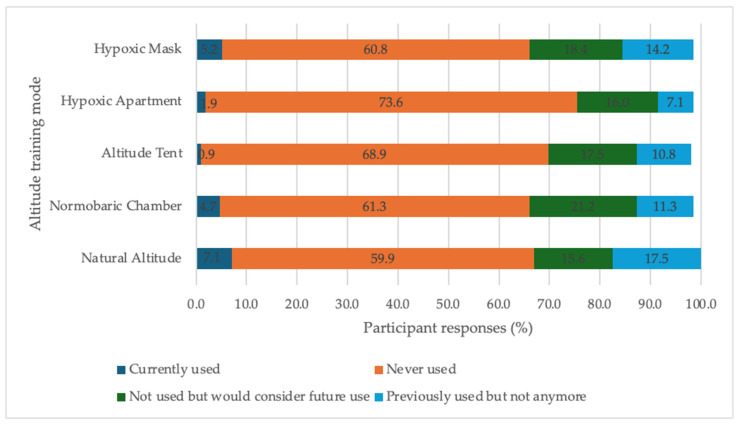
Participant response rate for various modes of hypoxic training.

**Figure 3:**
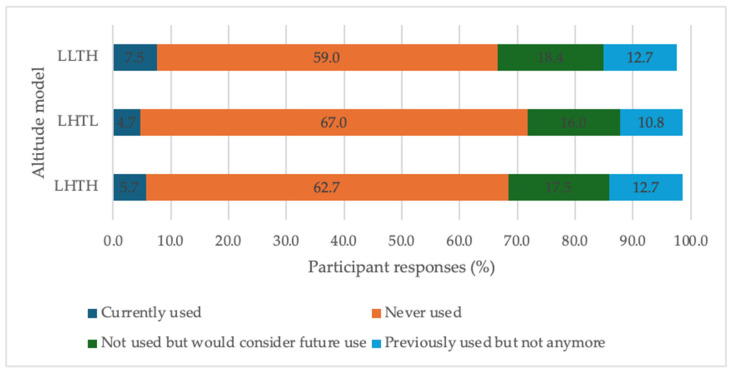
Participant response rate for the primary models of hypoxic training. LLTH = live low-train high, LHTL = live high-train low, LHTH = live high-train high.

Natural altitude (7.1%) was the most commonly reported mode, followed by hypoxic masks (5.2%) and normobaric chambers (4.7%), whereas hypoxic apartments (1.9%) and altitude tents (0.9%) were rarely used (Figure 2). Training models, including live low–train high (LLTH, 7.5%), live high–train high (LHTH, 5.7%), and live high–train low (LHTL, 4.7%), were reported at similarly low frequencies (Figure 3).

**Figure 4:**
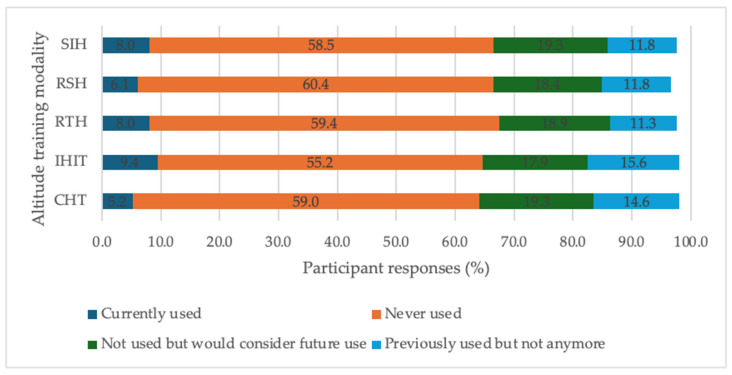
Participant response rate for a range of hypoxic training modalities. SIH = sprint interval training in hypoxia, RSH = repeated-sprint training in hypoxia, RTH = resistance training in hypoxia, IHIT = high-intensity interval training in hypoxia, CHT = continuous training in hypoxia.

High-intensity modalities in hypoxia, including IHIT (9.4%), resistance training in hypoxia (8%), and sprint interval training in hypoxia (8%), were more commonly reported than continuous hypoxic training (5.2%) and repeated-sprint training in hypoxia (6.1%) (Figure 4). Among participants who engaged in hypoxic training, exposure was typically limited to 1–2 phases per year (20.8%), lasting 1–2 weeks (10.4%), with 1–2 sessions per week (13.7%), generally conducted at ≤1500 m (13.7%) for up to 1 h per session (8.5%) (Table 3).

### 3.3. Education and Understanding

Most participants reported no formal education on hypoxic training (79.2%). Among those who had received education, the primary sources were sport coaches (19.3%), followed by other athletes (9.9%), sport scientists (7.5%), physiotherapists (7.1%), and strength and conditioning coaches (6.1%). Formal pathways such as university education (1%), school education (0.5%) and self-directed study (0.5%) were reported less frequently.

The most common sources of information were informal, including other people (22.2%), online searches (20.8%), and videos (19.8%). In contrast, more formal sources such as books (11.8%), magazines (8%), and expert consultation (0.5%) were used less frequently (Table 4).

### 3.4. Hypoxic Training Perceptions of Judo Athletes and Performance Staff

Participants generally perceived hypoxic training as demanding, with 50% agreeing it is hard and only 16% reporting it as enjoyable. Some also perceived hypoxic training to have psychological benefits, including improved focus (17%) and reduced exposure to life stressors (14.6%).

Perceptions of performance benefits were mixed, with some participants agreeing that hypoxic training may improve judo performance (38.7%), enhance fitness (30.2%), and support preparation for competition at altitude (37.7%), with fewer endorsing its role in injury rehabilitation (18.4%).

Descriptively, a greater proportion of performance staff than judo athletes reported positive perceptions of hypoxic training for judo performance (51.3% vs. 35.8%), fitness enhancement (43.6% vs. 27.2%), altitude competition preparation (56.4% vs. 33.5%) and improved focus (38.5% vs. 14.5%). Perceptions of its role in injury rehabilitation and reducing home-life stressors were similar across groups (Table 5).

### 3.5. Views on Benefits and Drawbacks of Hypoxic Training for Judo

Thematic analysis identified two higher-order themes: Hypoxic Training Benefits and Hypoxic Training Drawbacks, comprising 11 sub-themes and 22 codes (Supplementary File S6).

### 3.6. Hypoxic Training Benefits

Five sub-themes were identified: fitness and physiological enhancement, improved health and recovery, enhanced sport performance, psychological benefits, and others. The key themes are presented below.

### 3.7. Fitness and Physiological Enhancement

Participants frequently perceived hypoxic training as beneficial for improved fitness and physiological adaptation. Perceived benefits included enhanced endurance, increased red blood cell production, and time-efficient training adaptations, reflected in responses such as:

‘Same improvements of fitness can be achieved by less training time’

‘Higher levels of fitness, better endurance, shorter period of time to build fitness’

‘Increase in red blood cells’

### 3.8. Enhanced Sport Performance

Hypoxic training was also perceived to support judo performance, often through improvements in cardiovascular fitness:

‘Improve my performance in judo’

‘I think altitude training in general would improve my cardiovascular fitness and therefore my judo performance’

### 3.9. Psychological Benefits

Participants perceived hypoxic training to have some psychological benefits, including increased mental resilience and confidence:

‘Mental resilience’

‘Improved mental strength’

‘Increased levels of fitness drives belief’

### 3.10. Hypoxic Training Drawbacks

Six sub-themes were identified: adverse physiological changes, negative health effects, training impairment, psychological and cognitive effects, lifestyle barriers, and others. The key themes are presented below.

### 3.11. Lifestyle Barriers

Practical constraints were commonly reported as negative perceptions, particularly cost and access:

‘Facilities not always accessible for all athletes’

‘It would be very costly and unfeasible’

### 3.12. Training Impairment

Participants perceived hypoxic training as challenging when attempting to integrate hypoxic training within existing schedules, with concerns about disruption to judo and strength and conditioning training:

‘Restricted judo training can be carried out’

‘Difficult to fit into a busy training calendar to get a training block’

‘It is hard, and the timing and periodization needs to be carefully selected for optimal performance gains. Getting this wrong can be really detrimental’

### 3.13. Negative Health Effects

Potential adverse health effects were also perceived by participants, particularly during initial exposure:

‘Altitude sickness’

‘Adverse side effects, muscle loss and weight loss during the initial adaptation period’

‘Possible adverse reactions such as vertigo, vomiting and asthenia’

## 4. Discussion

The primary aim of this study was to examine hypoxic training practices, perceptions, and educational pathways among judo athletes and performance staff. The key findings were that: (1) hypoxic training is not widely used in judo; (2) when used, it is primarily aimed at enhancing sea-level performance and supporting injury rehabilitation; (3) perceptions of hypoxic training were mixed with some athletes and performance staff perceiving hypoxic training as potentially beneficial for fitness and judo performance; (4) most participants reported limited formal education on hypoxic training.

These findings align with the contemporary use of hypoxic training to enhance sea-level performance [5,6,7] and support injury rehabilitation [51]. In contrast, its traditional role in preparing athletes for competition at altitude [2,3,4] was less frequently reported (8.5%), likely reflecting the predominantly low-altitude context of judo competitions.

The preference for improving sea-level performance may also explain the relatively greater use of LLTH reported amongst respondents in the questionnaire, which has gained popularity in the literature for its potential to enhance performance and facilitate rehabilitation within shorter exposure periods [19,20,51]. Whilst evidence for hypoxic training in return-to-play and injury rehabilitation is limited [52], a notable proportion of respondents in the current study reported that they currently (13.2%) or will potentially use hypoxic training for injury recovery in the future (20.3%). Narrative evidence suggests that hypoxic training may allow for a reduced external load whilst maintaining physiological stimulus, with potential benefits for recovery [51].

Preliminary support for this concept comes from case-based research in judoka, where combined resistance and running training in normobaric hypoxia were associated with improvements in asymmetry, aerobic capacity, and hormonal responses [53]. However, such findings are based on single-case designs without control conditions and should be interpreted with caution. Furthermore, due to the explorative nature of the current study, only views and perceptions on the use of hypoxic training were assessed. Therefore, experimental research is required to establish the efficacy of hypoxic training in rehabilitation settings.

Despite the limited number of studies in judoka, which have largely been conducted at natural altitude [25,26,27,28], research in other combat sports has more commonly utilised simulated altitude approaches such as LLTH [54,55,56,57,58]. Many judo studies have been embedded within altitude training camps, whereas simulated approaches may better reflect typical training environments where access to natural altitude is constrained by geography, cost, and travel demands [20].

These practical constraints are consistent with participants’ responses in the present study, in which cost, access, and location were identified as key barriers perceived by respondents. However, natural altitude remained the most frequently reported mode (7.1%), which may reflect the geographical distribution of respondents and variable access to high-altitude environments.

High-intensity hypoxic training modalities (IHIT, RTH, SIH) were reported more frequently (8–9.4%) than lower-intensity approaches such as continuous hypoxic training (CHT; 5.2%). This broadly aligns with the existing literature, which suggests that high-intensity hypoxic methods, particularly maximal efforts, may offer greater performance benefits than submaximal approaches, for which the findings remain equivocal [20].

However, reported use of repeated-sprint training in hypoxia (RSH) was relatively underutilised (6.1%) compared with IHIT (9.4%), despite evidence supporting its effectiveness for improving high-intensity exercise performance [21,59]. This discrepancy may reflect practical constraints, familiarity, or differences in implementation rather than efficacy alone.

Emerging evidence in judo athletes suggests that RSH, including protocols using VHL, may improve repeated-sprint ability, potentially through enhanced muscle perfusion and maintenance of high-intensity fibre recruitment [21]. Nevertheless, the current evidence base remains limited, and further research is required to establish the effectiveness of RSH in combat sports.

Resistance training in hypoxia (RTH) was also reported by a proportion of participants (8%), which is consistent with the strong neuromuscular demands of judo [60]. Previous research in judoka has also explored RTH [25,27], reflecting its relevance for strength and power development. However, evidence comparing RTH with equivalent training in normoxia remains equivocal, likely due to variation in training protocols [61].

Local hypoxic methods, such as blood flow restriction (BFR) training, have shown potential to improve strength, hypertrophy, and endurance at lower loads [62,63,64], although the findings are not definitive. These approaches may be particularly relevant in load-compromised contexts, such as rehabilitation or periods of high training demand [65]. However, research examining their application in judo remains limited.

Reported hypoxic training exposure was relatively limited in the current study, with participants typically completing 1–2 phases per year (20.8%) lasting 1–2 weeks (10.4%), involving 1–2 sessions per week (13.7%) of approximately 1 h (8.5%) at ≤1500 m (8.5%). These patterns differ from those reported in endurance athletes, who more commonly undertake longer and more frequent hypoxic exposures at higher elevations [46]. In particular, the severity of altitude (i.e., elevation) is on the lower side of what would be considered training at altitude [66]. Generally, higher elevations are required in order to elicit robust physiological responses. Research in endurance athletes suggests altitudes of ~1800–2500 m are required for a prolonged period of time (>12 h·day^−1^) to see worthwhile changes to haemoglobin mass (a key indicator of improved oxygen transport) [18,19]. In addition, the duration of exposure reported by participants in the current study reflects the greater application of the LLTH model, given the fact that greater durations of exposure are required to see improvements in oxygen-carrying capacity with the traditional models as discussed. With that being said, the elevation reported would still likely be insufficient for seeing substantial non-haematological changes. LLTH research typically adheres to protocols exposing athletes to moderate-extreme altitudes (~2500–5500 m) [20]. Therefore, the use of proper education around the topic of hypoxic training may be a viable strategy for athletes considering implementing this type of training into their programme.

The discrepancy between hypoxic training practices in endurance sports and combat sports likely reflects differences in sport demands and training priorities. Hypoxic training is well established in endurance sports, where physiological adaptations such as increased capillarisation and myoglobin content are key determinants of performance [23,67]. In contrast, evidence supporting its application in combat sports remains limited. Accordingly, further research is needed to evaluate the effectiveness and practical relevance of hypoxic training in judo and similar disciplines.

From an educational perspective, most participants (79.2%) reported no formal education on hypoxic training. This may help explain the low levels of reported use, alongside qualitative responses indicating a desire for more sport-specific guidance (e.g., ‘keen on info specific to judo’). While there appears to be interest in hypoxic training, its application may be limited by uncertainty around its relevance and implementation in judo.

Education was primarily sourced informally, with coaches (19.3%), other individuals (22.2%), online searches (20.8%), and videos (19.8%) reported more frequently than formal sources such as sport scientists, academic materials, or structured courses. This suggests that knowledge in this area is largely socially transmitted and practice-led rather than formally structured.

Rather than reflecting a deficit in practice, this pattern may indicate the absence of judo-specific frameworks to guide the application of hypoxic training. In this context, practitioners appear to rely on a combination of experiential knowledge and available evidence. Future work should aim to develop sport-specific guidance that integrates scientific evidence with practical expertise to support informed decision-making in applied settings.

Overall, participants reported mixed perceptions of hypoxic training; some respondents perceived hypoxic training as effective for improving judo performance (38.7%), enhancing fitness (30.2%), and preparing for competition at altitude (37.7%), with fewer endorsing its role in injury rehabilitation (18.4%). Descriptively, performance staff expressed more favourable views than judo athletes across performance, fitness, and preparation outcomes. These differences may reflect variation in experience, education, or exposure to hypoxic training practices.

These perceptions were consistent with the qualitative findings, where participants perceived hypoxic training as a time-efficient strategy to improve fitness (e.g., “same improvements in fitness can be achieved with less training time”) and physiological adaptations (e.g., “increase in red blood cells”). At the same time, practical constraints—including cost, access, scheduling challenges, and potential health risks (e.g., “altitude sickness”)—were commonly reported perceptions. Similar barriers have been identified in endurance athletes and support staff [46]. Importantly, these findings reflect belief systems and experiential knowledge rather than evidence of effectiveness. In the absence of sport-specific research, such perceptions are likely to influence decision-making around the use and implementation of hypoxic training in judo.

This is the first study to examine hypoxic training practices, perceptions, and educational pathways in judo. The findings highlight clear differences between current practices in judo and recommendations largely derived from endurance and team sports, likely reflecting the distinct demands and constraints of combat sport. These results provide a foundation for future research. One potential direction is the development of a conceptual framework to guide the application of hypoxic training in combat sports, similar to approaches adopted in swimming and team sports [68,69]. Such work may help identify relevant performance determinants and support the development of more ecologically valid, sport-specific guidelines. Given the descriptive nature of the current study, these findings only provide preliminary insights and are based on participants’ perceptions rather than objective evidence supporting the use of hypoxic training for improving performance and injury rehabilitation in judo athletes. Therefore, future studies employing rigorous experimental research designs are required to evaluate the efficacy of hypoxic training for judo.

## 5. Strengths and Limitations

A strength of this study is the inclusion of both athletes (*n* = 173) and performance staff (*n* = 39), many operating at national and international levels, providing insight into applied practice in high-performance settings. These findings provide a useful insight into current hypoxic training practices among respondents, especially national and international-level participants, but should not be treated as fully representative of all judo athletes and performance staff. Finally, despite no formal psychometric validation, the questionnaire design was informed by previous research and refined through pilot testing, supporting clarity, readability, and contextual relevance of the questionnaire.

However, several limitations should be acknowledged. First, due to the voluntary nature of participating in this questionnaire, self-selection bias may have occurred as athletes and performance staff who are more interested in altitude or who have actually participated at altitude previously may have been more likely to respond to the questionnaire, thus becoming less representative of the target population. The reliance on self-reported items throughout the questionnaire may also result in recall bias as participants are self-reporting what they can remember about potential hypoxic practices they have participated in, for example, recalling the level of altitude they have previously trained at. Due to the convenience sampling strategy employed in this study and the international connections the authors have, there was a clear imbalance between the country response rate, with most responses coming from East Asia, South America and Europe, with a lack of responses from other countries. Although several translated versions were used to increase accessibility, not all versions underwent full forward-back translation or cross-cultural validation, so some variation in interpretation of altitude-related terminology may have occurred. Despite the study being adapted from previous research, checked independently by experts in the field and going through pilot testing, formal validation of the scale was not completed. Although key terminology was explained at the start of the questionnaire to mitigate the risk of confusion, it is possible that participants may have interpreted different altitude-related terms differently, resulting in variability between respondents. The study focused solely on judo, limiting generalisability to other combat sports. Additionally, the convenience sampling strategy used to recruit participants for the questionnaire may not have been considered representative of the overall judo population, as there was a clear discrepancy between male and female respondents (148 vs. 64, respectively) and between respondent level (204 international/national vs. 8 regional/club). Furthermore, the questionnaire did not capture local (e.g., blood flow restriction training) or passive (i.e., resting hypoxic exposure) hypoxic methods, which may also be relevant in practice. Future research should consider broader combat sport populations and a wider range of hypoxic modalities to provide a more comprehensive understanding of their application. Finally, the current study reported data using descriptive statistics exclusively; thus, no inferential statistics were used to make comparisons between athlete and performance staff responses. Future research should consider implementing inferential statistics to allow for a deeper analysis of the differences between athlete and performance staff responses.

## 6. Summary and Conclusions

Findings indicate that hypoxic training is not widely used in judo, and though perceptions of hypoxic training efficacy were mixed amongst judo athletes and performance staff, some respondents perceived hypoxic training as potentially beneficial for fitness and performance. However, these perceptions should not be interpreted as evidence of effectiveness. These findings are to be expected given the physical and physiological differences between judo and other endurance sports, such as running, which have a greater breadth of literature supporting its use in addition to traditionally being ingrained within the culture. When implemented, it is primarily aimed at enhancing sea-level performance rather than competition at altitude, likely reflecting the environments in which judo competitions typically take place. Formal education on hypoxic training appears limited, which may influence its application in practice. Findings from this study may help identify areas where sport-specific guidance and future experimental research are needed. However, given the explorative nature of this questionnaire and the lack of experimental research in judo, sport-specific recommendations are still required. Therefore, future research should consider using findings from this study as a catalyst for more investigative intervention-based research with the goal of developing a conceptual framework for judo-specific hypoxic training recommendations.

## Figures and Tables

**Table 1:** Participant characteristics.

	Judo Athlete (*n* = 173)	Performance Staff (*n* = 39)
Sex	M: 120 F: 53	M: 28 F: 11
Age (years)	21 ± 4	38 ± 10
Judo experience (years)	11 ± 5	13 ± 7
Competitive level	C: 0 R: 6 N: 90 I: 77	C: 1 R: 1 N: 14 I: 23

M = male, F = female, C = club, R = regional, N = national, I = international.

**Table 2:** Geographical location of the participants.

Country	Judo Athlete	Performance Staff
Austria	*n* = 0	*n* = 1
Brazil	*n* = 44	*n* = 11
Estonia	*n* = 1	*n* = 0
Great Britain	*n* = 16	*n* = 12
Ireland	*n* = 0	*n* = 1
Jamaica	*n* = 1	*n* = 0
Japan	*n* = 5	*n* = 3
Netherlands	*n* = 0	*n* = 1
Poland	*n* = 0	*n* = 1
Puerto Rico	*n* = 2	*n* = 0
Slovenia	*n* = 0	*n* = 1
South Korea	*n* = 103	*n* = 6
Spain	*n* = 1	*n* = 0
Not specified	*n* = 0	*n* = 2

**Table 3:** Hypoxic training parameters reported by judo athletes and performance staff.

Hypoxic Training Parameter	Response Option	Full Sample *n* (%)	Applicable Sample *n* (%)
Hypoxic training phases per year	Not applicable	141 (66.5%)	—
	1–2	44 (20.8%)	44 (62.0%)
	3–4	12 (5.7%)	12 (16.9%)
	5–6	7 (3.3%)	7 (9.9%)
	≥7	6 (2.8%)	6 (8.5%)
	Not sure	1 (0.5%)	1 (1.4%)
	Applicable total	71 (33.5%)	71 (100%)
Altitude height	Not applicable	145 (68.4%)	—
	≤1500 m	18 (8.5%)	18 (26.9%)
	1501–2000 m	10 (4.7%)	10 (14.9%)
	2001–2600 m	5 (2.4%)	5 (7.5%)
	2601–3200 m	2 (0.9%)	2 (3.0%)
	Not sure	32 (15.1%)	32 (47.8%)
	Applicable total	67 (31.6%)	67 (100%)
Phase duration	Not applicable	168 (79.2%)	—
	1–2 weeks	22 (10.4%)	22 (50.0%)
	3–4 weeks	10 (4.7%)	10 (22.7%)
	5–6 weeks	8 (3.8%)	8 (18.2%)
	≥7 weeks	4 (1.9%)	4 (9.1%)
	Applicable total	44 (20.8%)	44 (100%)
Sessions per week	Not applicable	170 (80.2%)	—
	1–2	29 (13.7%)	29 (69.0%)
	3–4	4 (1.9%)	4 (9.5%)
	5–6	2 (0.9%)	2 (4.8%)
	≥7	7 (3.3%)	7 (16.7%)
	Applicable total	42 (19.8%)	42 (100%)
Session duration	Not applicable	160 (75.5%)	—
	≤1 h	18 (8.5%)	18 (34.6%)
	1 h 1 min–2 h	15 (7.1%)	15 (28.8%)
	2 h 1 min–3 h	13 (6.1%)	13 (25.0%)
	3 h 1 min–4 h	3 (1.4%)	3 (5.8%)
	>4 h	1 (0.5%)	1 (1.9%)
	Applicable total	52 (24.5%)	52 (100%)

Note: the applicable sample varies by question depending on the number of participants responding to that specific question.

**Table 4:** Participant response rate to different education providers and sources.

* Education Provider	**Responses**
Sport scientists	16 (7.5%)
Sport coaches	41 (19.3%)
Strength and conditioning coaches	13 (6.1%)
Physiotherapists	15 (7.1%)
Athletes	21 (9.9%)
University education	2 (1%)
School education	1 (0.5%)
Self-studied	1 (0.5%)
Never used	3 (1.4%)
* Education Source	**Responses**
Journal articles	30 (14.2%)
Google	44 (20.8%)
Magazines	17 (8%)
Experts	1 (0.5%)
Other people	47 (22.2%)
Videos	42 (19.8%)
Books	25 (11.8%)
Not applicable	1 (0.5%)
None	1 (0.5%)

* Participants were able to select multiple responses for these questions.

**Table 5:** Participant perceptions on the various uses of hypoxic training in judo.

Hypoxic Training Perception Statement	Strongly Agree	Agree	Neither Agree nor Disagree	Disagree	Strongly Disagree
Training at altitude helps me prepare for competition at altitude	J: 17 (9.83%) PS: 5 (12.82%)	J: 41 (23.7%) PS: 17 (43.59%)	J: 91 (52.6%) PS: 15 (38.46%)	J: 9 (5.2%) PS: 0 (0%)	J: 7 (4.05%) PS: 1 (2.56%)
Altitude training has helped me recover from injuries	J: 5 (2.89%) PS: 1 (2.56%)	J: 30 (17.34%) PS: 6 (15.38%)	J: 105 (60.69%) PS: 26 (66.67%)	J: 16 (9.25%) PS: 5 (12.82%)	J: 8 (4.62%) PS: 0 (0%)
I feel physically fitter after an altitude training phase	J: 6 (3.47) PS: 3 (7.69%)	J: 41 (23.7%) PS: 14 (35.9%)	J: 99 (57.23%) PS: 20 (51.28%)	J: 10 (5.78%) PS: 0 (0%)	J: 8 (4.62%) PS: 1 (2.56%)
Altitude training improves my judo performance	J: 17 (9.83%) PS: 3 (7.69%)	J: 45 (26.01%) PS: 17 (43.59%)	J: 86 (49.71%) PS: 18 (46.15%)	J: 9 (5.2%) PS: 0 (0%)	J: 6 (3.47%) PS: 0 (0%)
Training at altitude helps me avoid home-life stressors	J: 5 (2.89%) PS: 1 (2.56%)	J: 20 (11.56%) PS: 5 (12.82%)	J: 105 (60.69%) PS: 24 (61.54%)	J: 26 (15.03%) PS: 5 (12.82%)	J: 10 (5.78%) PS: 3 (7.69%)
Training at altitude helps me focus on my training	J: 5 (2.89%) PS: 1 (2.56%)	J: 20 (11.56%) PS: 14 (35.9%)	J: 107 (61.85%) PS: 20 (51.28%)	J: 21 (12.14%) PS: 1 (2.56%)	J: 13 (7.51%) PS: 2 (5.13%)
I enjoy training at altitude	J: 4 (2.31%) PS: 0 (0%)	J: 21 (12.14%) PS: 9 (23.08%)	J: 98 (56.65%) PS: 24 (61.54%)	J: 27 (15.61%) PS: 4 (10.26%)	J: 16 (9.25%) PS: 1 (2.56%)
Training sessions at altitude feel harder	J: 22 (12.72%) PS: 1 (2.56%)	J: 67 (38.73%) PS: 16 (41.03%	J: 63 (36.42%) PS: 17 (43.59%)	J: 6 (3.47%) PS: 3 (7.69%)	J: 8 (4.62%) PS: 1 (2.56%)

Note: Judo athletes’ and performance staff responses are reported descriptively only; no inferential statistics were conducted. Overall agreement was calculated by combining ‘agree’ and ‘strongly agree’ responses. J = judo athlete, PS = performance staff.

## Data Availability

Data may be made available by request to the lead researcher.

## Supplementary Materials

The following supporting information can be downloaded at: https://www.mdpi.com/article/10.3390/sports14070277/s1, File S1: Shows the questionnaire for the judo athletes (the questionnaire for the performance staff is exactly the same minus one question); File S2: Shows the cover letter that was sent to participants; File S3: Shows the poster that was advertised on social media for additional responses; File S4: Shows a Checklist for Reporting Results of Internet E-Surveys (CHERRIES); File S5: Shows a Checklist for Reporting of Survey Studies (CROSS); File S6: Shows a mind map from the thematic analysis of the responses to the open-ended questions.

## References

[1] Luks A.M., Ainslie P.N., Lawley J.S., Roach R.C., Simonson T.S. (2021). Ward, Milledge & West’s High Altitude Medicine and Physiology.

[2] Billaut F., Gore C.J., Aughey R.J. (2012). Enhancing Team-Sport Athlete Performance Is Training Relevant?. Sports Med..

[3] Lundby C., Millet G.P., Calbet J.A., Bärtsch P., Subudhi A.W. (2012). Does ‘altitude training’ increase exercise performance in elite athletes?. Br. J. Sports Med..

[4] Saunders P.U., Garvican-Lewis L.A., Chapman R.F., Périard J.D. (2019). Special Environments: Altitude and Heat. Int. J. Sport Nutr. Exerc. Metab..

[5] Bonetti D.L., Hopkins W.G. (2009). Sea-level Exercise Performance Following Adaptation to Hypoxia: A Meta-Analysis. Sports Med..

[6] Chapman R.F., Laymon Stickford A.S., Lundby C., Levine B.D. (2014). Timing of Return from Altitude Training for Optimal Sea Level Performance. J. Appl. Physiol..

[7] Constantini K., Wilhite D.P., Chapman R.F. (2017). A Clinician Guide to Altitude Training for Optimal Endurance Exercise Performance at Sea Level. High Alt. Med. Biol..

[8] Haider T., Casucci G., Linser T., Faulhaber M., Gatterer H., Ott G., Linser A., Ehrenbourg I., Tkatchouk T., Burtscher M. (2009). Interval hypoxic training improves autonomic cardiovascular and respiratory control in patients with mild chronic obstructive pulmonary disease. J. Hypertens..

[9] Mackenzie R., Maxwell N., Castle P., Brickley G., Watt P. (2011). Acute hypoxia and exercise improve insulin sensitivity (S(I) (2*)) in individuals with type 2 diabetes. Diabetes/Metab. Res. Rev..

[10] Lyamina N.P., Lyamina S.V., Senchiknin V.N., Mallet R.T., Downey F.H., Manukhina E.B. (2011). Normobaric hypoxia conditioning reduces blood pressure and normalizes nitric oxide synthesis in patients with arterial hypertension. J. Hypertens..

[11] Hayes H.B., Jayaraman A., Herrmann M., Mitchell G.S., Rymer W.Z., Trumbower R.D. (2014). Daily intermittent hypoxia enhances walking after chronic spinal cord injury: A randomized trial. Neurology.

[12] Pramsohler S., Burtscher M., Faulhaber M., Gatterer H., Rausch L., Eliasson A., Netzer N.C. (2017). Endurance Training in Normobaric Hypoxia Imposes Less Physical Stress for Geriatric Rehabilitation. Front. Physiol..

[13] Heinicke K., Heinicke I., Schmidt W., Wolfarth B. (2005). Three-Week Traditional Altitude Training Increases Hemoglobin Mass and Red Cell Volume in Elite Biathlon Athletes. Int. J. Sports Med..

[14] Garvican-Lewis L.A., Halliday I., Abbiss C.R., Saunders P.U., Gore C.J. (2015). Altitude Exposure at 1800 m Increases Haemoglobin Mass in Distance Runners. J. Sports Sci. Med..

[15] Sitkowski D., Szygula Z., Pokrywka A., Turowski D., Malczewska-Lenczowska J. (2018). Interrelationships between changes in erythropoietin, plasma volume, haemoglobin concentration, and total haemoglobin mass in endurance athletes. Res. Q. Exerc. Sport.

[16] Hoppeler H., Vogt M. (2001). Muscle tissue adaptations to hypoxia. J. Exp. Biol..

[17] Gore C.J., Clark S.A., Saunders P.U. (2007). Nonhematological mechanisms of improved sea-level performance after hypoxic exposure. Med. Sci. Sports Exerc..

[18] Flaherty G., O’Connor R., Johnston N. (2016). Altitude training for elite endurance athletes: A review for the travel medicine practitioner. Travel Med. Infect. Dis..

[19] McLean B.D., Gore C.J., Kemp J. (2014). Application of ‘Live Low-Train High’ for Enhancing Normoxic Exercise Performance in Team Sport Athletes. Sports Med..

[20] Girard O., Brocherie F., Goods P.S.R., Millet G.P. (2020). An Updated Panorama of “Living Low-Training High” Altitude/Hypoxic Methods. Front. Sports Act. Living.

[21] Faiss R., Raberin A., Brocherie F., Millet G.P. (2025). Repeated-sprint training in hypoxia: A review with 10 years of perspective. J. Sports Sci..

[22] Khodaee M., Grothe H.L., Seyfert J.H., VanBaak K. (2016). Athletes at High Alititude. Sports Health.

[23] Mujika I., Sharma A.P., Stellingwerff T. (2019). Contemporary Periodization of Altitude Training for Elite Endurance Athletes: A Narrative Review. Sports Med..

[24] Girard O., Amann M., Aughey R., Billaut F., Bishop D.J., Bourdon P., Buchheit M., Chapman R., D’Hooghe M., Garvican-Lewis L.A. (2013). Position statement—Altitude training for improving team-sport players’ performance: Current knowledge and unresolved issues. Br. J. Sports Med..

[25] Almeida F., Padial P., Bonitch-Góngora J.P., de la Fuente B., Schoenfeld B.J., Morales-Artacho A.J., Benavente C., Feriche B. (2021). Effects of Power-Oriented Resistance Training During an Altitude Camp on Strength and Technical Performance of Elite Judokas. Front. Physiol..

[26] Tomazin K., Almeida F., Stirn I., Padial P., Bonitch-Góngora J., Morales-Artacho A.J., Strojnik V., Feriche B. (2021). Neuromuscular Adaptations after an Altitude Training Camp in Elite Judo Athletes. Int. J. Environ. Res. Public Health.

[27] Almeida F., Bonitch-Góngora J., Feriche B., Schoenfeld B.J., de la Fuente B., Padial P. (2022). Altitude differentially alters the force-velocity relationship after 3 weeks of power-oriented resistance training in elite judokas. Eur. J. Sport Sci..

[28] Purnamasari I., Novian G., Febrianty M.F., Rismayadi A. (2024). Endurance training for judo athletes: Improving anaerobic and aerobic capacity in the high altitude. J. Sport Area.

[29] Franchini E., Artioli G.G., Brito C.J. (2013). Judo combat: Time-motion analysis and physiology. Int. J. Perform. Anal. Sport.

[30] Coswig V.S., Gentil P., Bueno J.C.A., Follmer B., Marques V.A., Del Vecchio F.B. (2018). Physical fitness predicts technical-tactical and time-motion profile in simulated Judo and Brazilian Jiu-Jitsu matches. PeerJ.

[31] Sacripanti A., Ahmedov F. (2021). Judo combat: Time-motion analysis and biomechanical approach. Arts Sci. Judo.

[32] Julio U.F., Panissa V.L., Esteves J.V., Cury R.L., Agostinho M.F., Franchini E. (2017). Energy-system contributions to simulated judo matches. Int. J. Sports Physiol. Perform..

[33] Serrano M.A., Salvador A., González-Bono E., Sanchis C., Suay F. (2001). Relationships between recall of perceived exertion and blood lactate concentration in a judo competition. Percept. Mot. Ski..

[34] Degoutte F., Jouanel P., Filaire E. (2003). Energy demands during a judo match and recovery. Br. J. Sports Med..

[35] Laskowski R., Kujach S., Smaruj M., Grzywacz T., Luszczyk M., Marek A., Ziemann E. (2012). Lactate concentration during one-day male judo competition: A case study. Arch. Budo.

[36] Detanico D., Dal Pupo J., Franchini E., dos Santos G.S. (2012). Relationship of aerobic and neuromuscular indexes with specific actions in judo. Sci. Sports.

[37] Franchini E., Miarka B., Matheus L., Del Vecchio F.B. (2011). Endurance in Judogi grip strength tests: Comparison between elite and non-elite judo players. Arch. Budo.

[38] Kons R.L., da Silva Athayde M.S., da Silva Junior J.N., Katcipis L.F.G., Detanico D. (2020). PREDICTORS OF JUDO-SPECIFIC TASKS FROM NEUROMUSCULAR PERFORMANCE IN YOUNG ATHLETES AGED 11-16 YEARS. Int. J. Sports Phys. Ther..

[39] Faiss R., Léger B., Vesin J.M., Fournier P.E., Eggel Y., Deriaz O., Millet G.P. (2013). Significant molecular and systemic adaptations after repeated sprint training in hypoxia. PLoS ONE.

[40] Beard A., Ashby J., Chambers R., Brocherie F., Millet G.P. (2019). Repeated-sprint training in hypoxia in international rugby union players. Int. J. Sports Physiol. Perform..

[41] Birol A., Aras D., Akalan C., Aldhahi M.I., Gulu M. (2024). Three sessions of repeated sprint training in normobaric hypoxia improves sprinting performance. Heliyon.

[42] Woorons X., Faucher C., Dufour S.P., Brocherie F., Robach P., Connes P., Brugniaux J.V., Verges S., Gaston A.F., Millet G. (2024). Hypoventilation training including maximal end-expiratory breath holding improves the ability to repeat high-intensity efforts in elite judo athletes. Front. Physiol..

[43] Michalczyk M., Czuba M., Zydek G., Zajac A., Langfort J. (2016). Dietary Recommendations for Cyclists during Altitude Training. Nutrients.

[44] Bärtsch P., Swenson E.R. (2013). Acute high-altitude illnesses. N. Engl. J. Med..

[45] Bhattacharjee B., Azhar S., Kansal K.J., Kuriyal S., Mishra D.K., Tanigaiselvane D.J., Calyanasundaram M. (2025). Effect of Sleep Disturbance at High Altitude Training Camps on Young Athletes’ Performance. J. Carcinog..

[46] Turner G., Fudge B.W., Pringle J.S.M., Maxwell N.S., Richardson A.J. (2019). Altitude training in endurance running: Perceptions of elite athletes and support staff. J. Sports Sci..

[47] Chyung S.Y., Roberts K., Swanson I., Hankinson A. (2017). Evidence-based survey design: The use of a midpoint on the Likert scale. Perform. Improv..

[48] Eysenbach G. (2004). Improving the quality of Web surveys: The Checklist for Reporting Results of Internet E-Surveys (CHERRIES). J. Med. Internet Res..

[49] Sharma A., Minh Duc N.T., Luu Lam Thang T., Nam N.H., Ng S.J., Abbas K.S., Huy N.T., Marusic A., Paul C.L., Kwok J. (2021). A Consensus-Based Checklist for Reporting of Survey Studies (CROSS). J. Gen. Intern. Med..

[50] Braun V., Clarke V. (2006). Using thematic analysis in psychology. Qual. Res. Psychol..

[51] Narang B.J., Drole K., Barber J.F.P., Goods P.S.R., Debevec T. (2024). Utility of hypoxic modalities for musculoskeletal injury rehabilitation in athletes: A narrative review of mechanisms and contemporary perspectives. J. Sports Sci..

[52] Yeung W.C.V., Kwok V., Ihsan M., Girard O. (2025). Hypoxia Conditioning for Load-Compromised Athletes: A Narrative Review Exploring Potential Applications in Injury and Disability Management. Sports Med..

[53] Drozd M., Lubon W., Turpin J.A.P., Grzyb W. (2025). The Influence of Step Load Periodisation Based on Time Under Tension in Hypoxic Conditions on Hormone Concentrations and Postoperative ACL Rehabilitation of a Judo Athlete: A Case Study. J. Clin. Med..

[54] Ambrozy T., Maciejczyk M., Klimek A.T., Wiecha S., Stanula A., Snopkowski P., Palka T., Jaworski J., Ambrozy D., Rydzik L. (2020). The effects of intermittent hypoxic training on anaerobic and aerobic power in boxers. Int. J. Environ. Res. Public Health.

[55] Torrealba T.C., Araya J.A., Benoit N., Deldicque L. (2020). Effects of high-intensity interval training in hypoxia on taekwondo performance. Int. J. Sports Physiol. Perform..

[56] Hagiwara M., Yamagishi T., Okamoto S., Azuma Y., Yamashita D. (2023). Short-term repeated sprint training in hypoxia improves explosive power production capacity and repeated sprint ability in Japanese international-level male fencers: A case study. Physiol. Rep..

[57] Ambrozy T., Snopkowski P., Rydzik L., Kedra A., Wasacz W. (2025). The impact of the experimental “Hypoxic Boxing” training on the motor abilities and specialized fitness of national boxing champions: A randomized controlled trial. Front. Physiol..

[58] Baurzhan M., Ten A., Kulbayev A., Shepetyuk M., Myrzabossynov Y., Nassiyev Y., Komarov O., Zhidovinova A., Nurtazina J., Absattarova K. (2025). Impact of intermittent hypoxic training on haematological, biochemical, and functional parameters in Qazaq Kuresi Wrestlers: A pilot study. J. Phys. Educ. Sport.

[59] Faiss R., Girard O., Millet G.P. (2013). Advancing hypoxic training in team sports: From intermittent hypoxic training to repeated sprint training in hypoxia. Br. J. Sports Med..

[60] Monteiro L., Massuca L.M., Ramos S., Garcia-Garcia J. (2024). Neuromuscular Performance of World-Class Judo Athletes on Bench Press, Prone Row and Repeated Jump Tests. Appl. Sci..

[61] Benavente C., Schoenfeld B.J., Padial P., Feriche B. (2023). Efficacy of resistance training in hypoxia on muscle hypertrophy and strength development: A systematic review with meta-analysis. Sci. Rep..

[62] Gronfeldt B.M., Lindberg Nielsen J., Mieritz R.M., Lund H., Aagaard P. (2020). Effect of blood-flow restricted vs. heavy-load strength training on muscle strength: Systematic review and meta-analysis. Scand. J. Med. Sci. Sports.

[63] Geng Y., Wu X., Zhang Y., Zhang M. (2024). Potential Moderators of the Effects of Blood Flow Restriction Training on Muscle Strength and Hypertrophy: A Meta-analysis Based on a Comparison with High-Load Resistance Training. Sports Med..

[64] de Lemos Muller C.H., Ramis T.R., Ribeiro J.L. (2019). Effects of low-load resistance training with blood flow restriction on the perceived exertion, muscular resistance and endurance in healthy young adults. Sport Sci. Health.

[65] Perera E., Ming Z.X., Nolan S.H., Bedi A., Ayeni O.R., Khan M. (2022). Effects of Blood Flow Restriction Therapy for Muscular Strength, Hypertrophy, and Endurance in Healthy and Special Populations: A Systematic Reivew and Meta-Analysis. Clin. J. Sport Med..

[66] Bärtsch P., Saltin B. (2008). General introduction to altitude adaptation and mountain sickness. Scand. J. Med. Sci. Sports.

[67] van der Zwaard S., Brocherie F., Jaspers R.T. (2021). Under the Hood: Skeletal Muscle Determinants of Endurance Performance. Front. Sports Act. Living.

[68] Truijens M.J., Rodriguez F.A. (2010). Altitude and Hypoxic Training in Swimming. World Book of Swimming: From Science to Performance.

[69] Bishop D.J., Girard O. (2013). Determinants of team-sport performance: Implications for altitude training by team-sport athletes. Br. J. Sports Med..

