# Supplementary material for: Hypoxic Training for Judo: Practices, Perceptions and Education of Judo Athletes and Performance Staff
Source: Sports (Basel). 2026 Jul 3;14(7):277. doi: 10.3390/sports14070277 (PMC13417016; doi:10.3390/sports14070277)
Supplement: Supplementary file 1 [file sports-14-00277-s001.zip › sports-4349966-supplementary.pdf]

Supplementary Files

Informed Consent

You have been invited to participate in an online questionnaire study, titled- *Hypoxic Training for Judo: Practices, Perceptions and Education of Judo Athletes and Performance Staff.* The following study will be conducted by Joshua Till in collaboration with the University of Wolverhampton. The questionnaire includes questions around your experience with altitude training, your understanding of its use and how effective you believe it to be. You are free to decline participation in this study and withdraw from the study at any point.
Data from this study may be used for further publication. However, any data provided in this questionnaire will remain anonymous, confidential and will be protected under the Data Protection Act (2018). Finally, this research has been approved by the University of Wolverhampton ethics committee.

If you have any questions or queries about this study, please email me using the following email address-j.e.till@wlv.ac.uk.

Supervisory team: Associate Professor Ross Cloak r.cloak@wlv.ac.uk and Professor Andrew Lane a.m.lane2@wlv.ac.uk

* Indicates required question

1. I confirm that I am at least 18 years old, have read and understood the above and agree to participate in this study

Mark only one oval.

- ◯ Yes
- ◯ No

Altitude Training for Judo

Welcome to the questionnaire and thank you for agreeing to take part in this research. Two frequently used terms in this questionnaire are altitude and hypoxic. Altitude is how high you are above sea-level and hypoxic refers to a lack of oxygen in the air. Other specialist terminology will be defined throughou the questionnaire to aid your understanding.
Please ensure that you answer all questions as honestly as possible as there are no right or wrong answers. With all that out of the way, I hope you enjoy the questionnaire and thank you for your time!

Located between Nepal and Tibet, Mount Everest claims the greatest altitude on earth of 8,849 r above sea-level at the peak of the mountain.

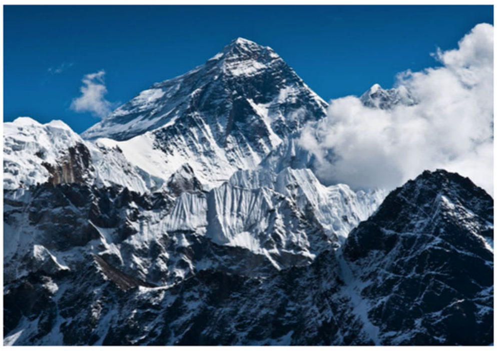

Participant Information

This first set of questions relate to general information of you as an athlete. To answer these question: please
select the suitable option for multiple choice questions and type in the space provided below for any short answer questions. On some of the multiple choice questions the option to type an answer is alsc possible where "Other..." is provided.

2. Which gender do you compete under?

Mark only one oval.

- Male
- Female

3. What is your age? (minimum age for this questionnaire is 18)

4. What country do you represent?

5. How long have you trained in judo for?

6. What is the highest level you have competed at in judo?

Mark only one oval.

- International
- National
- Regional
- Club
- Other: ____________

Altitude Practices

This next section relates to your use and experience with altitude/hypoxic training. To answer these questions please identify how frequently from "Never used" too "Not used but would consider future use" for the below options. For any
multiple choice questions please select the suitable option(s)

and for the final question of this section please type in the space provided.

7. Use of altitude/hypoxic training?

Mark only one oval per row.

	Never used	Previously used but not anymore	Currently used	Not used but would consider future use
**Preparing for competition at altitude**				
**Preparing for competition at sea-level**				
**Improving sea-level performance**				
**Aiding return to play/injury rehabilitation**				

8. Altitude living/training methods?

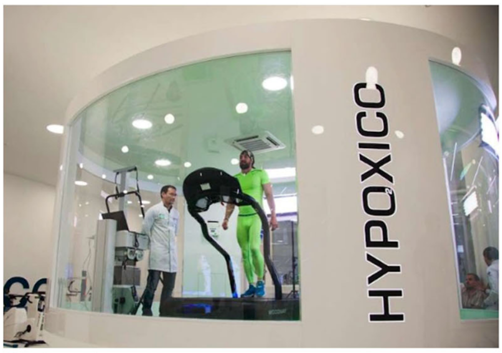

Mark only one oval per row.

	Never used	Previously used but not anymore	Currently used	Not used but would consider future use
**Natural altitude (living and/or training in mountanious areas well above sea-level)**				
**Normobaric chamber (a room that manually reduces the oxygen content breathed in)**				
**Altitude tent**				
**Hypoxic apartment**				
**Hypoxic mask**				

9. Models of training?

Mark only one oval per row.

	Never used	Previously used but not anymore	Currently used	Not used but would consider future use
Live high-train high (living and training at altitude i.e. 2000-2500 m)	◯	◯	◯	◯
Live high-train low (living at altitude i.e. 2000-2500 m and training near or at sea-level i.e. less than 1500 m)	◯	◯	◯	◯
Live low-train high (living at sea-level and training at altitude i.e. 2500-3500 m)	◯	◯	◯	◯

10. Hypoxic training modes?

Mark only one oval per row.

	Never used	Previously used but not anymore	Currently used	Not used but would consider future use
Continuous training in hypoxia	◯	◯	◯	◯
Interval training in hypoxia	◯	◯	◯	◯
Resistance training in hypoxia	◯	◯	◯	◯
Repeated-sprint training in hypoxia	◯	◯	◯	◯
Sprint intervals in hypoxia	◯	◯	◯	◯

11. How many times per year do you participate in altitude/hypoxic training phases?

Mark only one oval.

- ◯ Not applicable
- ◯ 1-2
- ◯ 3-4
- ◯ 5-6
- ◯ 7 or more

12. What altitude/simulated altitude (a low oxygen environment caused artificially by using: chambers, apartments, tents and masks) do you live and/or train at? (select all answers th apply)

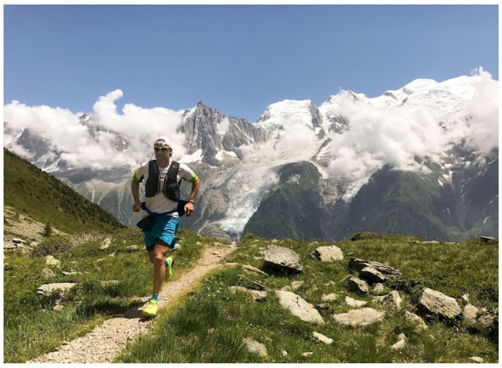

Check all that apply.

- ☐ Not applicable
- ☐ Up to and including 1,500 m
- ☐ 1,501-2,000 m
- ☐ 2,001-2,600 m
- ☐ 2,601-3,200 m
- ☐ Over 3,201 m
- ☐ Not sure

13. How many weeks do you live and/or train at altitude/simulated altitude for? (select all answers that apply)

Check all that apply.

- ☐ Not applicable
- ☐ 1-2
- ☐ 3-4
- ☐ 5-6
- ☐ 7 or more

14. How many sessions per week do you train at altitude/simulated altitude? (select all answe that apply)

Check all that apply.

- ☐ Not applicable
- ☐ 1-2
- ☐ 3-4
- ☐ 5-6
- ☐ 7 or more

15. How many hours per day do you spend living and/or training at altitude/simulated altitude (select all answers that apply)

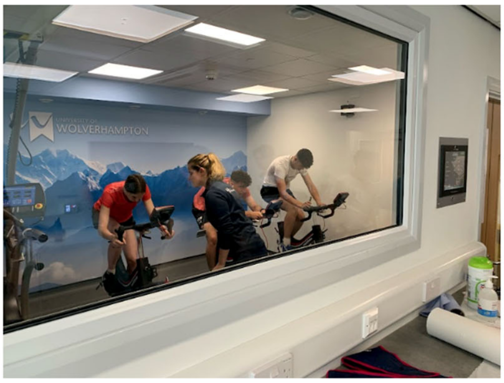

Check all that apply.

- [ ] Not applicable
- [ ] Up to and including 1 hour
- [ ] 1 hour 1 minute - 2 hours
- [ ] 2 hours 1 minute - 3 hours
- [ ] 3 hours 1 minute - 4 hours
- [ ] Over 4 hours 1 minute

16. In a few short sentences please provide the main reasons as to why you chose the above answers to the questions (for example, why did you choose "Not used but would consider future use" for live high-train low?)

____
____
____
____
____

Education and Understanding

This set of questions covers how you have been educated in relation to altitude/hypoxic training and your current understanding of its use. To answer these questions please select the suitable option(s) for multiple choice questions and type in the space provided for long answer questions. On some of the multiple choice questions the option to type an answer is also possible where "Other..." is provided.

17. Have you previously been educated on altitude/hypoxic training?

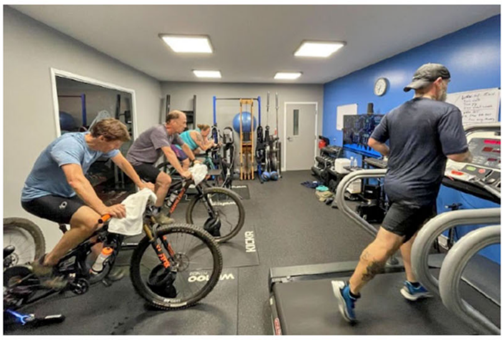

Mark only one oval.

- ( ) Yes
- ( ) No

18. Who have you been you educated by? (select all answers that apply)

Check all that apply.

- [ ] Sport coaches
- [ ] Strength and conditioning coaches
- [ ] Physiotherapists
- [ ] Sport scientists
- [ ] Athletes
- [ ] Other: ____________________

19. What other sources of education have you used? (select all answers that apply)

Check all that apply.

- [ ] Magazines
- [ ] Google
- [ ] Videos
- [ ] Journal articles
- [ ] Books
- [ ] Other people
- [ ] Other: ____________________

20. State three benefits of altitude/hypoxic training (in order of importance)

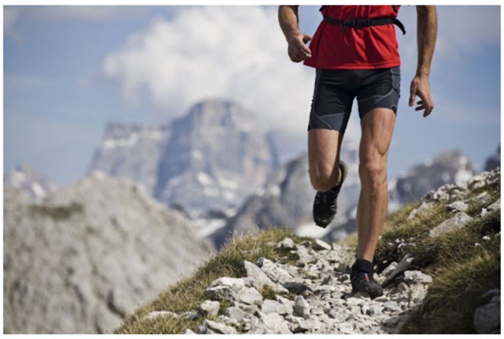

____
____
____
____
____

21. State three drawbacks of altitude/hypoxic training (in order of importance)

____
____
____
____
____

Altitude Perceptions

The final section of this questionnaire covers your perceptions of altitude/hypoxic training and its effectiveness.
For this section please identify how much you "Agree" or "Disagree" with the below statements:

22. Training sessions at altitude/simulated altitude feel harder

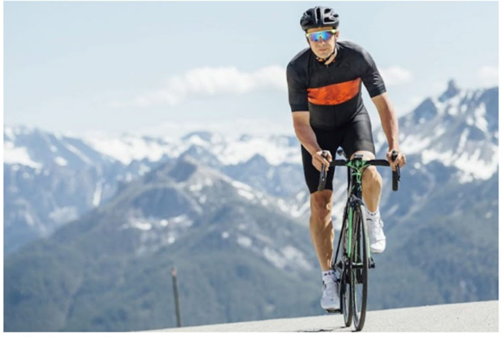

Mark only one oval.

- ◯ Strongly disagree
- ◯ Disagree
- ◯ Neither agree nor disagree
- ◯ Agree
- ◯ Strongly agree

23. I enjoy training at altitude/simulated altitude

Mark only one oval.

- ◯ Strongly disagree
- ◯ Disagree
- ◯ Neither agree nor disagree
- ◯ Agree
- ◯ Strongly agree

24. Training at altitude/simulated altitude helps me focus on my training

Mark only one oval.

- ◯ Strongly disagree
- ◯ Disagree
- ◯ Neither agree nor disagree
- ◯ Agree
- ◯ Strongly Agree

25. Training at altitude/simulated altitude helps me avoid home-life stressors

Mark only one oval.

- ◯ Strongly disagree
- ◯ Disagree
- ◯ Neither agree nor disagree
- ◯ Agree
- ◯ Strongly agree

26. Altitude/simulated altitude training improves my judo performance

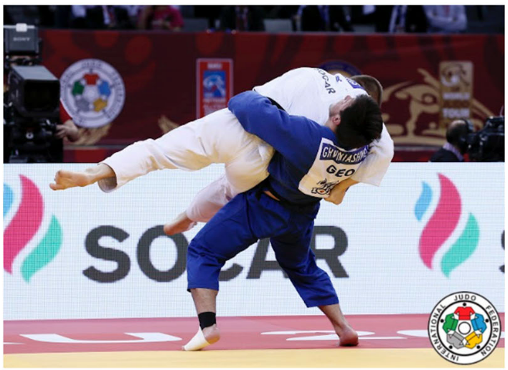

Mark only one oval.

- ◯ Strongly disagree
- ◯ Disagree
- ◯ Neither agree nor disagree
- ◯ Agree
- ◯ Strongly agree

27. I feel physically fitter after an altitude/hypoxic training phase

Mark only one oval.

- Strongly disagree
- Disagree
- Neither agree nor disagree
- Agree
- Strongly agree

28. Altitude/simulated altitude training has helped me recover from injuries

Mark only one oval.

- Strongly disagree
- Disagree
- Neither agree nor disagree
- Agree
- Strongly Agree

29. Training at altitude/simulated altitude helps me prepare for competitions at altitude

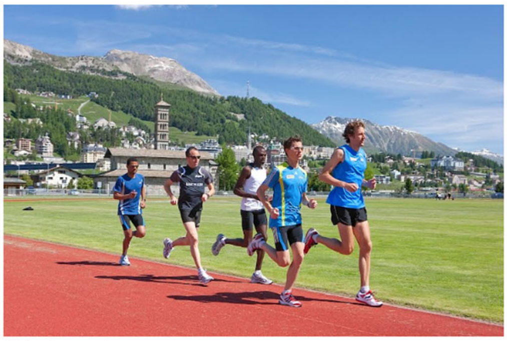

Mark only one oval.

- Strongly disagree
- Disagree
- Neither agree nor disagree
- Agree
- Strongly agree

Welcome to the end of the questionnaire!

Thank you for time and effort in completing this questionnaire. The information collected from this questionnaire will help to provide a valuable insight into the current practices, understanding and perceptions of altitude training amongst elite judokas.

Please check out the below link for access to this study once published:
https://www.researchgate.net/profile/Joshua_Till/research

Please click on the purple "submit" button below to send over the questionnaire results.

This content is neither created nor endorsed by Google.

Google Forms

File S1 Shows the questionnaire for the judo athletes (the questionnaire for the performance staff is exactly the same minus one question).

UNIVERSITY OF
WOLVERHAMPTON

Dear athlete/coach/practitioner,

You are invited to participate in a study conducted by Joshua Till, a PhD student from the University of Wolverhampton. The purpose of this study is to explore the altitude practices, perceptions and educational methods used and implemented by elite judo athletes and coaches/practitioners. Should you choose to participate in this study, your contribution will assist in providing evidence-based guidelines for altitude training prescription for judo athletes and may help to guide future research.

This study requires the completion of an online questionnaire that consists of up to 28 questions and should take approximately 10-15 minutes to complete. Due to the nature of this research, there are no known risks associated with taking part in the study. The decision to participate in this study is your own and you are free to withdraw or reject participation at any time without further follow-up.

If you do choose to participate in this research, please ensure that you read the informed consent at the start of the questionnaire and select "yes" to proceed with the questionnaire. All data provided in this study will remain strictly confidential and will conform with the University of Wolverhampton research data management policies and the Data Protection Act (2018). Additionally, to ensure anonymity, any personal information will be securely stored away from any information that could be used to identify you as the participant.

If you have any further questions or queries about this study, please do not hesitate to get in contact with me using the following email address- j.e.till@wlv.ac.uk. Finally, I want to thank you again for taking time to read through this letter and I hope to hear from you soon.

If you are an athlete and are interested in completing the questionnaire, please click on the following link- https://fm.addxt.com/form/?vf=1FAIpQLSeccItmuir4SpupgVAxFpThEy5ctMjjGyyGydf8-P2k2npnOQ

If you are a coach/practitioner who is interested, please click on this link to access the questionnaire- https://fm.addxt.com/form/?vf=1FAIpQLSebyxItW7PP0g9439pw4g_iJMzQ0QuoLvUfPaAjkk9mwxCmFQ

Your sincerely,

Joshua Till

Supervisory team: Associate Professor Ross Cloak r.cloak@wlv.ac.uk and Professor Andrew Lane a.m.lane2@wlv.ac.uk

File S2 Shows the cover letter that was sent to participants.

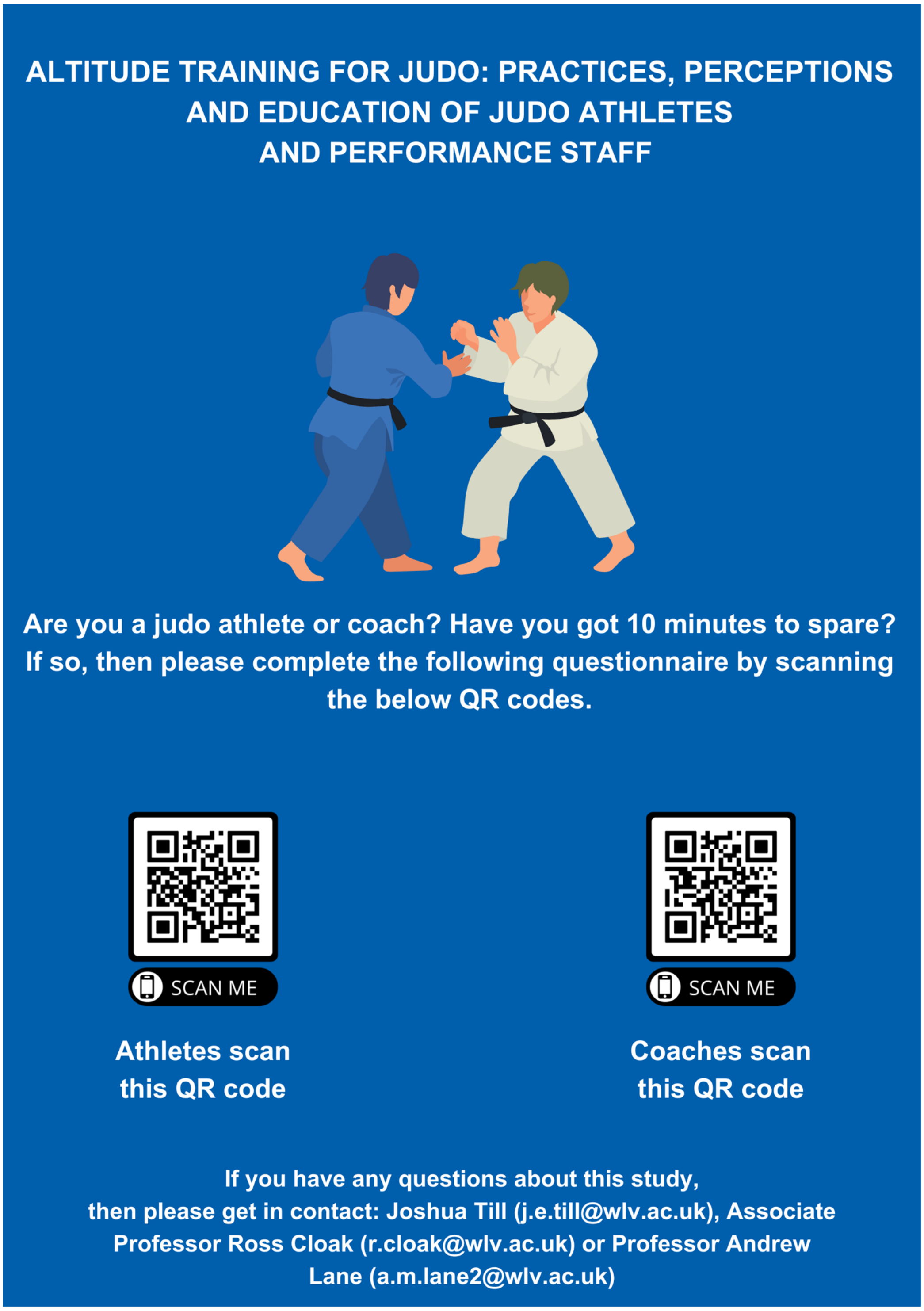

File S3 Shows the poster that was advertised on social media for additional responses.

Item Category	Checklist Item	Explanation
**Design**	Describe survey design	A cross-sectional survey study was conducted using anonymous online questionnaires to examine altitude training practices, perceptions, and educational pathways among judo athletes and performance staff.
**IRB (Institutional Review Board) approval and informed consent process**	IRB approval	Yes. The study was approved by the University of Wolverhampton ethics committee.
	Informed consent	Before accessing the questionnaire, participants received a cover letter explaining the study's purpose. The questionnaire included an informed consent section before participation.
	Data protection	The survey was administered online anonymously via Google Forms. Responses were exported for data cleaning and preparation, and the anonymous design supported the confidentiality of participants' information.
**Development and pre-testing**	Development and testing	The questionnaires were adapted from previous research in endurance athletes (Turner et al., 2019). Two versions were developed, one for judo athletes and one for performance staff, with minor terminology differences. After review by the research team, the questionnaires were piloted on four performance staff members and four athletes from the British Judo National Team to assess content validity, readability, and visual appeal. Minor adjustments were made to the wording and order of the questions.
**Recruitment process and description of the sample having access to the questionnaire**	Open survey versus closed survey	Open survey. The questionnaire was distributed through professional networks, social media platforms, international contacts, and in-person recruitment.
	Contact mode	Participants were contacted via email through organizations such as British Judo and the International Judo Federation, as well as through social media and in-person distribution.
	Advertising the survey	The questionnaire was advertised through professional organizations, international research/practice connections (e.g., Brazil, Japan, and Korea), and social

		media platforms, including Instagram, LinkedIn, and X. It was also dispersed in person.
**Survey administration**	Web/E-mail	The questionnaires were administered online using Google Forms, and the survey link was distributed by email and social media.
	Context	The survey was self-administered by judo athletes and performance staff from multiple countries, including South Korea, Brazil, the United Kingdom, Japan, and others.
	Mandatory/voluntary	Voluntary.
	Incentives	No incentives were provided for participants taking part in this study.
	Time/Date	Responses were collected from January 2024 to January 2025.
	Randomization of items or questionnaires	Questions were presented in a fixed order to align with the separate sections in the questionnaire.
	Adaptive questioning	Adaptive questioning was not used in this study. However, two separate questionnaires were used for athletes and performance staff, with one question omitted for staff because it was not relevant.
	Number of Items	The final questionnaire comprised 29 questions for judokas and 28 questions for performance staff.
	Number of screens (pages)	There were 7 pages for both questionnaires.
	Completeness check	Not explicitly reported. The manuscript states that responses were exported for data cleaning and preparation, but no detailed description of automated completeness checks within the platform was provided.
	Review step	Participants were able to review questions or change responses by clicking the back button at the bottom of the page.
**Response rates**	Unique site visitor	Not applicable / not reported.
	View rate (Ratio of unique survey visitors/unique site visitors)	Not applicable / not reported.
	Participation rate (Ratio of unique visitors who agreed	Not reported.

	to participate / unique first survey page visitors)	
	Completion rate (Ratio of users who finished the survey / users who agreed to participate)	243 potential participants responded to the questionnaire. 30 were excluded as they were under 18 and another 1 as they had never done judo before. This left 212 participants who completed the study questionnaire and were included within the analysis.
**Preventing multiple entries from the same individual**	Cookies used	No cookies were used for this questionnaire.
	IP check	IP addresses were not used for identification of participants.
	Log file analysis	No other techniques were used to analyse log files.
	Registration	This questionnaire utilised an open access design and thus, no registration was required.
**Analysis**	Handling of incomplete questionnaires	Incomplete questionnaires from participants (e.g. missed some questions) were included within the analysis. However, individual missing responses to questions were excluded (i.e. if a participant missed a question, their response was not taken into consideration for the findings for that question).
	Questionnaires submitted with an atypical timestamp	No timeframe cut-off was set for participants to complete the questionnaire in.
	Statistical correction	No statistical correction for weighting or representativeness was reported. Closed-ended responses were analysed descriptively using frequency distributions in SPSS, and open-ended responses were analysed using reflexive thematic analysis in NVivo.

File S4 Shows a Checklist for Reporting Results of Internet E-Surveys (CHERRIES)

Section/topic	Item	Item description	Completed	Notes
**Title and abstract**	1a	State the word “survey” along with a commonly used term in the title or abstract to introduce the study’s design.	Yes	The abstract states that this was a cross-sectional survey.
	1b	Provide an informative summary in the abstract, covering background, objectives, methods, findings/results, interpretation/discussion, and conclusions.	Partially	The abstract includes introduction, methods, results, and conclusions, but requires minor revision due to repetition and formatting issues.
**Introduction**	2	Provide a background about the rationale of the study, what has been previously done, and why this survey is needed.	Yes	The introduction contextualizes altitude training, its physiological basis, and highlights the lack of research on altitude training in judo compared to endurance and team sports.
	3	Identify specific purposes, aims, goals, or objectives of the study.	Yes	The three aims (practices, perceptions, and education) are clearly stated at the end of the introduction.
**Methods**	4	Specify the study design in the methods section with a commonly used term (e.g., cross-sectional or longitudinal).	Yes	The study is described as a cross-sectional survey.
	5a	Describe the questionnaire (e.g., number of sections, number of questions, number and names of instruments used).	Yes	The questionnaire comprised 5 sections: 29 questions for athletes and 28 for performance staff.
**Data collection methods**	5b	Describe all questionnaire instruments that were used in the survey to measure particular concepts.	Yes	The questionnaire covered participant characteristics, altitude practices, education, and perceptions. It was adapted from previous research (Turner et al., 2019).
	5c	Provide information on the questionnaire's pretesting.	Yes	The questionnaire was piloted with four performance staff

				members and four athletes from the British Judo National Team, resulting in minor adjustments.
	5d	The questionnaire should be fully provided (appendix or supplement).	Yes	The questionnaire is listed in Appendix A.
**Sample characteristics**	6a	Describe the study population (background, location, eligibility criteria).	Yes	Participants included 212 individuals (judokas and performance staff) from multiple countries, with inclusion criteria clearly described.
	6b	Describe sampling techniques used.	Yes	The sampling technique administered for this study was convenience sampling. Recruitment was achieved via professional networks, social media, and in-person contact.
	6c	Provide information on sample size and sample size calculation.	Partially	Sample size (n = 212) is reported, but no priori sample size calculation was conducted.
	6d	Describe how representative the sample is of the target population.	Yes	Representativeness is acknowledged as a limitation of the current study.
**Survey administration**	7a	Provide information on modes of questionnaire administration.	Yes	The survey was administered online via Google Forms and distributed via email, social media, and in-person recruitment.
	7b	Provide information on survey time frame.	Yes	Responses were collected from January 2024 to January 2025.
	7c	Provide information on preventing multiple participation.	No	No technical procedures (e.g., IP checks, cookies) for preventing multiple participation were used.
**Study preparation**	8	Describe preparation process before conducting the survey.	Yes	Questionnaire development, expert review, piloting, and

				recruitment strategies are described.
**Ethical considerations**	9a	Provide information on ethical approval and informed consent.	Yes	Ethics approval from the University of Wolverhampton is reported, and informed consent was obtained online.
	9b	Provide information about anonymity and confidentiality.	Yes	A statement on anonymity and confidentiality is provided at the start of the questionnaire within the informed consent.
**Statistical analysis**	10a	Describe statistical methods and software used.	Yes	Descriptive statistics were conducted in SPSS, and thematic analysis in NVivo.
	10b	Report modification of variables.	Yes	Nominal data such as gender was coded in SPSS to make analysis easier.
	10c	Report how missing data was handled.	No	Incomplete questionnaires from participants (e.g. missed some questions) were included within the analysis. However, individual missing responses to questions were excluded (i.e. if a participant missed a question, their response was not taken into consideration for the findings for that question).
	10d	State how the non-response error was addressed.	No	Not reported.
	10e	For longitudinal surveys, address loss to follow-up.	NA	The study is cross-sectional.
	10f	Indicate whether weighting or adjustment methods were used.	Yes	No weighting or representativeness adjustment was applied.
	10g	Describe any sensitivity analysis conducted.	No	No sensitivity analyses were reported.

**Results**	11a	Report the number of individuals at each stage of the study.	Yes	243 potential participants responded to the questionnaire. 30 were excluded as they were under 18 and another 1 as they had never done judo before. The remaining 212 participants were included in the analysis.
	11b	Provide reasons for non-participation.	No	Not reported.
	11c	Report response rate.	Yes	A total of 212 participants were included within the analysis.
	11d	Provide information on unique visitors and participation proportions.	No	Not reported.
**Descriptive results**	12	Provide characteristics of study participants.	Yes	Participant characteristics (sex, age, experience, country, level) are described.
**Main findings**	13a	Provide estimates with appropriate statistics.	Partially	Results are presented descriptively (percentages), without inferential comparisons.
	13b	Provide details for multivariable analysis.	NA	No multivariable analysis was conducted.
	13c	Provide details about the sensitivity analysis.	NA	Not applicable.
**Discussion**	14	Discuss limitations of the study.	Yes	Limitations include focus on judo only and omission of certain hypoxic methods. Additional limitations could be further expanded.
	15	Provide cautious interpretation and future directions.	Yes	The discussion provides cautious interpretation and highlights the need for sport-specific frameworks.
	16	Discuss external validity.	Yes	External validity is discussed in how the questionnaire findings can be applied to

				amateur and elite judo athletes.
**Other sections**	17	Role of funding source.	Yes	Funding for this study was provided by the University of Wolverhampton.
	18	Conflict of interest.	Yes	The authors declare that they do not have any conflict of interest.
	19	Acknowledgements.	Yes	The authors are thankful of British Judo for their support in this project which included distribution and initial advertisement of the survey. This work would also not have been possible without the support from the University of Wolverhampton.

File S5 Shows a Checklist for Reporting of Survey Studies (CROSS)

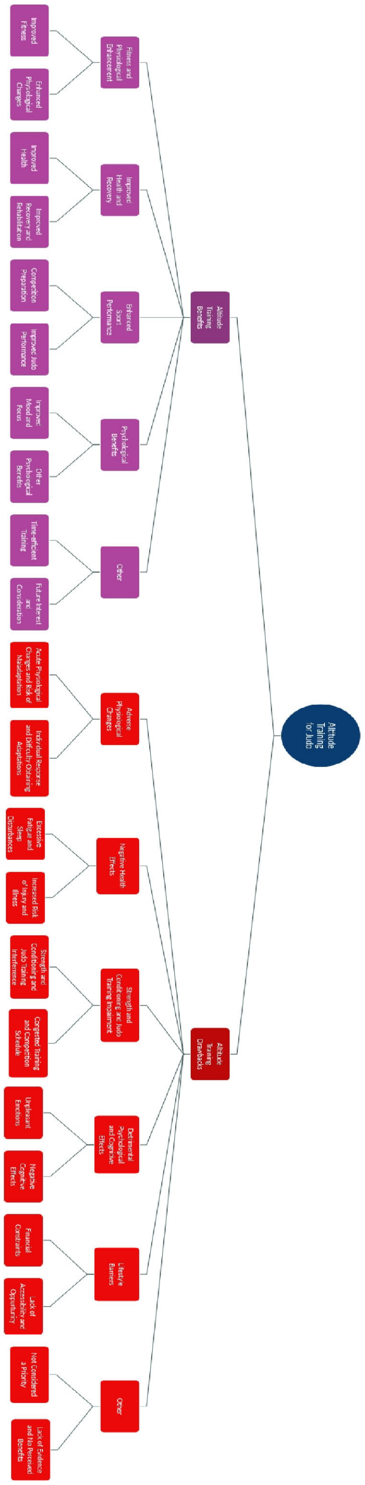

File S6 Shows a mind map from the thematic analysis of the responses to the open-ended questions.
